# TRIM32 Promotes the Growth of Gastric Cancer Cells through Enhancing AKT Activity and Glucose Transportation

**DOI:** 10.1155/2020/4027627

**Published:** 2020-01-21

**Authors:** Jianjun Wang, Yuejun Fang, Tao Liu

**Affiliations:** Department of Gastroenterological Surgery, Jinhua Guangfu Oncology Hospital, Huancheng North Road No. 1296, Jinhua, Zhejiang, China

## Abstract

Tripartite motif protein 32 (TRIM32), an E3 ubiquitin ligase, is a member of the TRIM protein family. However, the underlying function of TRIM32 in gastric cancer (GC) remains unclear. Here, we aimed to explore the function of TRIM32 in GC cells. TRIM32 was induced silencing and overexpression using RNA interference (RNAi) and lentiviral-mediate vector in GC cells, respectively. Moreover, the PI3K/AKT inhibitor LY294002 was used to examine the relationship between TRIM32 and AKT. Quantitative reverse-transcription PCR (qRT-PCR) and western blot were used to determine the mRNA and protein contents. The glucose analog 2-NBDG was used as a fluorescent probe for determining the activity of glucose transport. An annexin V-fluorescein isothiocyanate apoptosis detection kit was used to stain NCI-N87, MKN74, and MKN45 cells. Cell counting kit-8 (CCK-8) assay was used to examine cell proliferation. Our results indicated that TRIM32 was associated with poor overall survival of patients with GC. Moreover, TRIM32 was a proproliferation and antiapoptosis factor and involved in the AKT pathway in GC cells. Furthermore, TRIM32 possibly mediated the metabolism of glycolysis through targeting GLUT1 and HKII in GC cells. Importantly, TRIM32 silencing deeply suppressed the tumorigenicity of GC cells *in vivo*. Our findings not only enhanced the understanding of the function of TRIM32 but also indicated its potential value as a target in GC treatment.

## 1. Introduction

Gastric cancer (GC) is one of the most common tumors, which is the third leading cause of death-related cancer all over the world [1]. Although the surgical operation and chemotherapy contribute to GC patients, the outcome is far from being fully satisfied. Due to the high morbidity and mortality rates of GC, the novel effective therapies for GC are urgently needed [2]. Therefore, gaining a deep insight into the molecule mechanism of GC is a critical step for developing novel therapeutic approaches.

Tripartite motif protein 32 (TRIM32) belongs to the TRIM protein family, which is identified as an E3 ubiquitin ligase [3]. A previous report has demonstrated that TRIM32 is overexpressed in breast cancer and promotes the proliferation of breast cancer cells [4]. The similar results are also obtained in hepatocellular carcinoma cells [5]. Furthermore, TRIM32 inhibits the activity of PI3K/AKT/Foxo signaling in muscle atrophy through improving plakoglobin-PI3K dissociation [6]. However, the detailed function of TRIM32 in GC cells is less investigated.

The AKT signaling pathway plays an essential role in various biological functions, which is commonly abnormal in certain human cancer [7]. The phosphorylation of AKT is critical for its function. The inactivation of the PI3K/AKT pathway has induced G2/M arrest and apoptosis in lung cancer cells [8]. Previous evidence has demonstrated that enhancing the activity of AKT increases the chemoresistance for GC [9]. Moreover, PI3K/AKT also plays a key role in the apoptosis and proliferation of GC cells [10, 11]. However, the relationship between TRIM32 and AKT has not been confirmed yet in GC cells.

Glycolysis provides metabolic products and energy for cell survival. The activity of glycolysis is accelerated in certain human cancer cells [12]. A previous report has indicated that the glycolysis inhibitor not only prevents the tumor promoting effects but also shows the high value in cancer therapy treatment [13, 14]. Moreover, AKT has enhanced the activity of glycolysis in human liver and cervical tumor cells [15]. Furthermore, the PI3K/AKT signaling pathway is essential for suppressing glycolysis in oral squamous cell carcinoma cells [16]. Furthermore, the combined inhibitors of AKT and glycolysis showed a stronger effect in lung cancer treatment [17]. However, the precise function of TRIM32 in the glycolysis process remains unclear in GC cells.

The aim of the present study is to examine the function of TRIM32 and potential target in GC cells. RNA interference (RNAi) and lentiviral vector were used to silencing and overexpression TRIM32 in GC cells. Our data not only obtain a deep insight of the molecular function of TRIM32 in GC cells but also provide evidences to indicate its potential value as a target for GC treatment.

## 2. Methods and Materials

### 2.1. Cell Culture

The GC cell lines used in this study (NCI-N87, MKN74, HGC27, AGS, MKN45, and GES-1) were purchased from the cell bank of the Shanghai Biology Institute (Shanghai, China). Fetal bovine serum (10%) (GIBCO, USA) was added to all culture media along with 2 mM L-glutamine and 1% penicillin/streptomycin (Solarbio, China). Cells were grown in DMEM (Trueline, USA) and maintained in a 5% CO_2_ atmosphere at 37°C. The PI3K/AKT inhibitor LY294002 (Selleck, USA) was dissolved in DMSO. This study was approved by the ethics committee of Guangfu Hospital and followed the tenants of the Declaration of Helsinki. Moreover, there are no live subjects at the present study, so the informed consent is not necessary in this research.

### 2.2. RNA Isolation and Real-Time PCR

Total RNA from cell samples was extracted using TRIzol Reagent (Invitrogen, USA). Then, the cDNA synthesis kit (Fermentas, Canada) were used for the RNA reverse transcribed into complementary DNA (cDNA) according to the instructions of the manufacturer. The expression of GAPDH was used to normalize the gene expression and counted using the 2^−ΔΔCt^ method. Three replicates are needed for each analysis. The primers used in this study are presented in Supplementary [Supplementary-material supplementary-material-1].

### 2.3. Lentiviral-Mediated RNA Interference and Overexpression of TRIM32

Three plasmids containing siRNA targeting positions of human TRIM32 (NM_012210.3) were synthesized (Major, China). The negative control (siNC) contained a nonspecific scrambled siRNA sequence. A lentiviral plasmid (pLVX-puro) containing the full-length human TRIM32 cDNA sequence and an empty plasmid acted as negative controls (oeNC). Lipofectamine 2000 (Invitrogen, USA) was used to transiently transfect plasmids into cells according to the instructions of the manufacturer. Experiments were performed 48 h after the transfection. Detailed information on the sequence of siTRIM32s is provided in Supplementary [Supplementary-material supplementary-material-1].

### 2.4. Western Blot

Whole protein lysates were extracted from the indicated cells (NCI-N87, MKN74, and MKN45) using RIPA lysis buffer (JRDUN, Shanghai, China) with an EDTA-free protease inhibitor cocktail (Roche, Germany). An enhanced BCA protein assay kit (Thermo Fisher, USA) was utilized to estimate the protein concentration. Equal amounts of total protein (25 *μ*g) were fractionated using 10% SDS-PAGE and transferred to a nitrocellulose membrane (Millipore, USA) overnight. Then, after being blocked with 5% nonfat dry milk for 1 h at room temperature, the membranes were probed at 4°C overnight with the primary antibodies followed by incubation for 1 h at 37°C with the secondary antibody (anti-mouse IgG) (1 : 1000; Beyotime, China). An enhanced chemiluminescence system (Tanon, China) was used to detect the level of protein expression. Each sample was tested in triplicate, and GAPDH served as the internal reference. Detailed information on the primary antibodies is provided in Supplementary [Supplementary-material supplementary-material-1].

### 2.5. Cell Proliferation Assay

Cell counting kit-8 (CCK-8) assay (SAB, USA) was used to examine the cell proliferation according to the protocol of the manufacturer. Briefly, cells transfected as indicated were seeded in 96-well plates and cultured for 0, 24, 48, and 72 h, and then CCK-8 solution (1 : 10) was mixed into each well and incubated for 1 h. A microplate reader (Pulangxin, China) was used to measure the optical density values (OD) at a wavelength of 450 nm. Each time point was tested in triplicate.

### 2.6. Cell Apoptosis Assay

In brief, an annexin V-fluorescein isothiocyanate (FITC) apoptosis detection kit (Beyotime, China) was used to stain NCI-N87, MKN74, and MKN45 cells according to the instructions of the manufacturer at 48 h after viral infection. Then, a flow cytometer (BD, USA) was used to determine cells.

### 2.7. Glucose Transport and Lactate Production Assay

In brief, the glucose analog 2-NBDG (Molecular Probes, Eugene, OR) was used as a fluorescent probe for determining the activity of glucose transport. In order to examine the uptake of 2-NBDG, a total of 5 × 10^5^ cells from different groups were seeded in 6-well plates. Then, all the cells were preincubated in Krebs-Ringer bicarbonate (KRB) buffer (glucose free) for 15 min after maintaining in a 5% CO_2_ atmosphere at 37°C for 24 h. After that, cells incubated in fresh KRB buffer were supplemented with 2-NBDG for 45 min at 37°C, 5% CO_2_. Flow cytometry using a GloMax®-Multi + flow cytometer (Promega, USA) was used to quantitatively analyze the stained cells. Moreover, a Lactate Assay Kit (njjcbio, China) was utilized to examine the production of lactate in different cells according to the protocol of the manufacturer.

### 2.8. TUNEL Staining

TUNEL staining assays were performed with sections using a Tunel kit (11684817910, Roche, Switzerland) principally according to the supplier's instruction. Three replicates were needed for each sample.

### 2.9. Xenograft Model

The assay was carried out according to the Institute's guidelines for animal experiments and was approved by the independent ethics committee of Jin hua Guangfu Oncology Hospital, Jinhua, zhejiang, P.R. China, and and all animals were treated in accordance with the Institutional Animal Care and Use Committee (IACUC). An equal number of siNC- and siTRIM32-transfected NCI-N87 cells (*n* = 5 × 10^6^) were subcutaneously injected into the right flank of 4- to 6-week-old nude mice (*n* = 6 for each group; Shanghai Laboratory Animal Company, Shanghai. The length and width of the tumor were examined every 3 days for 33 days after being injected. The volume of the tumor was counted as length × (width 2/2). Six weeks after injection, six mice from the siNC- and siTRIM32-injected groups were sacrificed by cervical dislocation, and tumor tissues were fixed in 4% formalin for further analysis.

### 2.10. Statistical Analysis

GraphPad Prism software Version 7.0 (CA, USA) was utilized for the statistical analyses. Data were displayed as the mean ± SD of at least three samples. Statistical significance was determined by one-way analysis of variance (ANOVA) for multiple comparisons. Statistical significance was accepted by the *P* value <0.05.

## 3. Results

### 3.1. TRIM32 Upregulation was Associated with Poor Overall Survival of GC Patients

**Figure 1:**
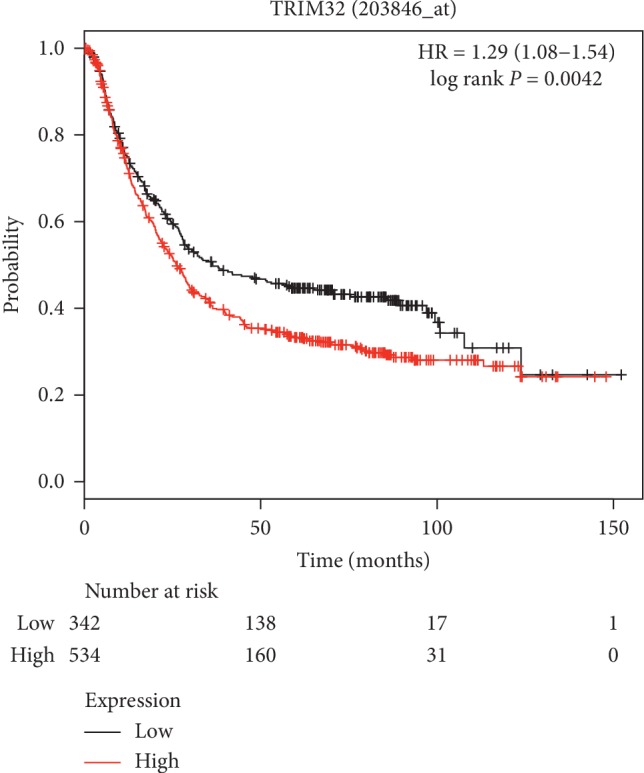
TRIM32 upregulation was associated with poor overall survival of GC patients.

To determine the function of TRIM32, one data set (ID: 203846_at) collected from gastric cancer database (http://kmplot.com) was used to quantify the connection between TRIM32 and overall survival (OS) of patients with GC. As presented in Figure 1, the OS of GC patients with high level of TRIM32 (*n* = 534) was much lower than that in GC patients with low-TRIM32 level (Figure 1). Thus, these results demonstrated that TRIM32 upregulation was associated with poor OS of GC patients.

### 3.2. The mRNA and Protein Level of TRIM32 in GC Cell Lines

**Figure 2:**
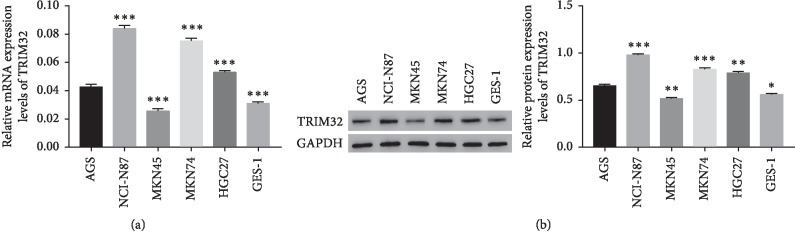
The relative mRNA and protein level of TRIM32 in different GC cells. (a) The relative mRNA level of TRIM32 in different GC cells. ^*∗∗∗*^*P* < 0.001 vs. AGS. (b) The relative protein level of TRIM32 in different GC cells. ^*∗∗∗*^*P* < 0.001 vs. AGS.

In this study, a total of six GC cell lines were used to examine the mRNA and protein level of TRIM32, including NCI-N87, MKN74, HGC27, AGS, MKN45, and GES-1. As shown in Figures 2(a) and 2(b), both the mRNA and protein of TRIM32 were significantly upregulated in NCI-N87 and MKN74 cells compared with that of AGS cells. Moreover, the level of TRIM32 was obviously downregulated in MKN45 cells among the GC cell lines as indicated above. Therefore, NCI-N87, MKN74, and MKN45 were chosen for knockdown and overexpression (OE) analysis.

### 3.3. Silencing and Overexpression of TRIM32 in GC Cells

**Figure 3:**
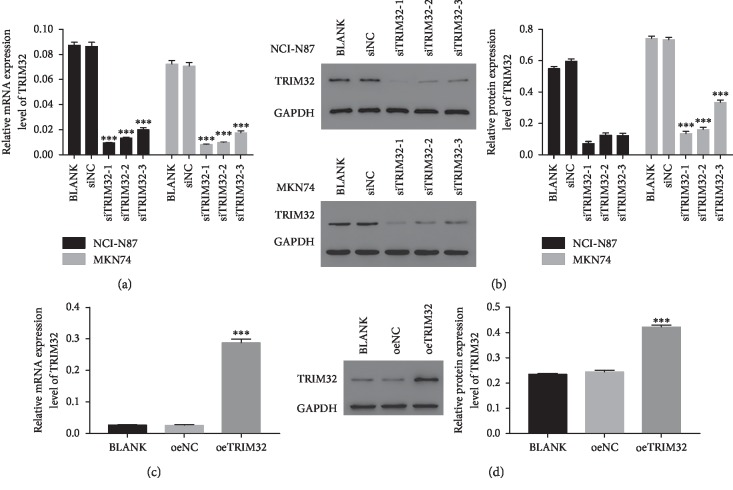
Knockdown and overexpression of TRIM32 in GC cells. (a) and (b) stand for the relative mRNA and protein level of NCI-N87 and MKN74 cells transfected with siNC, siTRIM32-1, siTRIM32-2, and siTRIM32-3, respectively. ^*∗∗∗*^*P* < 0.001 vs. siNC. (c) and (d) stand for the mRNA and protein level of oeTRIM32 transfected into MKN45 cells. ^*∗∗∗*^*P* < 0.001 vs. oeNC.

To silence the expression of TRIM32, three short interference RNAs (siRNAs) targeting human TRIM32 (siTRIM32-1, siTRIM32-2, and siTRIM32-3) and a nonspecific scrambled siRNA (siNC) were synthesized and transfected into NCI-N87 and MKN74 cell lines. The untreated cells acted as a blank control (BLANK). As shown in Figures 3(a) and 3(b), all three TRIM32-siRNAs strongly reduced the level of endogenous TRIM32. Moreover, RNAi1-1 and RNAi1-2 showed a stronger effect in inhibiting the expression of TRIM32 than RNAi1-3. Therefore, RNAi1-1 and RNAi1-2 were chosen for further study.

Moreover, MKN45 cells were transfected with a plasmid-overexpressing TRIM32 (oeTRIM32) and a mock plasmid (oeNC). Clearly, both the relative mRNA and protein level of TRIM32 were significantly upregulated in oeTRIM32-transfected cells (Figures 3(c) and 3(d)). Hence, the oeTRIM32-transfected cells were chosen for the following overexpression analysis.

### 3.4. TRIM32 siRNAs Inhibited the Proliferation and Induced the Apoptosis of GC Cells

**Figure 4:**
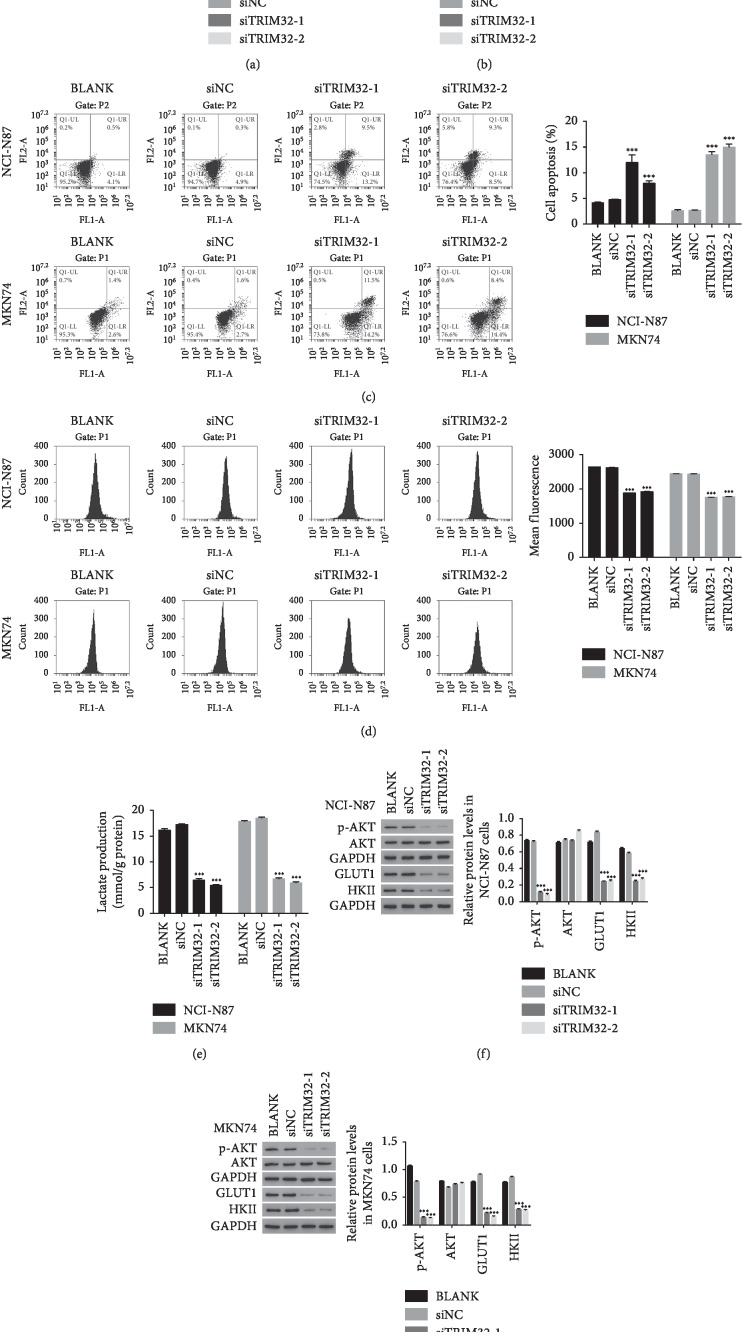
Knockdown of TRIM32 suppressed GC cells growth. (a) and (b) stand for cell proliferation that was detected 0, 24, 48, and 72 hours after transfection with siNC, siTRIM32-1, and siTRIM32-2 in NCI-N87 and MKN74 cells, respectively. ^*∗*^*P* < 0.05 vs. siNC, ^*∗∗∗*^*P* < 0.001 vs. siNC. (c) The apoptosis profile of siNC, siTRIM32-1, and siTRIM32-2 transfected into NCI-N87 and MKN74 cells, respectively. ^*∗∗∗*^*P* < 0.001 vs. siNC. (d) Glucose transport activity measured using the fluorescent glucose analog 2-NBDG in NCI-N87 and MKN74 cells transfected with siNC, siTRIM32-1, and siTRIM32-2, respectively. ^*∗∗∗*^*P* < 0.001 vs. siNC. (e) The production of lactate in NCI-N87 and MKN74 cells transfected with siNC, siTRIM32-1, and siTRIM32-2, respectively. ^*∗∗∗*^*P* < 0.001 vs. siNC. (f) and (g) stand for the protein level of AKT, p-AKT, GLUT1, and HKII in NCI-N87 and MKN74 cells transfected with siNC, siTRIM32-1, and siTRIM32-2, respectively. ^*∗∗∗*^*P* < 0.001 vs. siNC.

The Cell Counting Kit-8 (CCK-8) assay was performed to examine the function of siTRIM32s in the proliferation of GC cells. As shown in Figures 4(a) and 4(b), the cell proliferation rate was significantly suppressed in siTRIM32-transfected cells. Moreover, we also determined the function of siTRIM32s in the apoptosis of GC cells. Our results suggested that TRIM32 silencing remarkably improved the apoptosis of GC cells (Figure 4(c)). These results demonstrated that TRIM32 was a proproliferation and antiapoptosis factor in GC cells.

### 3.5. Glucose Transport Activity and Lactate Production was Decreased by siTRIM32s

We also determined the glucose transport activity and lactate production in TRIM32 siRNA transfected cells. As shown in Figure 4(d), the glucose transport activity showed no difference between BLANK and siNC transfected cells. However, the glucose transport activity was deeply decreased in NCI-N87 and MKN74 cells transfected with siTRIM32, respectively. Additionally, the lactate production also presented no difference between BLANK and siNC transfected cells. However, a remarkable downregulation of the lactate production was appeared in siTRIM32 transfected cells (Figure 4(e)). Taken together, these results reflected the possible function of TRIM32 in the energy metabolism of GC cells.

### 3.6. Silencing TRIM32 Suppressed the Glycolysis-Related Protein in GC Cells

Glucose transporter 1 (GLUT1) is an important regulator in the process of glucose uptake. Previous report has demonstrated that GLUT1 promotes the proliferation and metastasis of breast cancer cells [18]. Moreover, it has been reported that GLUT1 is involved in the invasion and metastasis of lung cancer cells [19]. Furthermore, Glycolytic enzyme Hexokinase II (HKII) has played an important role in tumor glycolysis and the progression of cancers. HKII is necessary for tumor initiation and maintenance [20]. Moreover, downregulating the expression of HKII contributes to suppress tumor glycolysis metabolism and tumor growth and induce apoptosis in cervical cancer cells [21].

In this study, western blot was used to determine the protein level of GLUT1 and HKII in different NCI-N87 and MKN74 transfected cells as indicated. Our results indicated that the protein contents of GLUT1 and HKII were downregulated in siTRIM32 transfected cells (Figures 4(f) and 4(g)). Therefore, TRIM32 might target GLUT1 and HKII in the regulation of glycolysis metabolism in GC cells. Interestingly, knockdown of TRIM32 significantly deeply inhibited the phosphorylation of AKT in a time-dependent manner in NCI-N87 and MKN74 ([Supplementary-material supplementary-material-1]).

### 3.7. The Function of TRIM32 Was Inhibited by a Specific AKT Inhibitor LY294002

**Figure 5:**
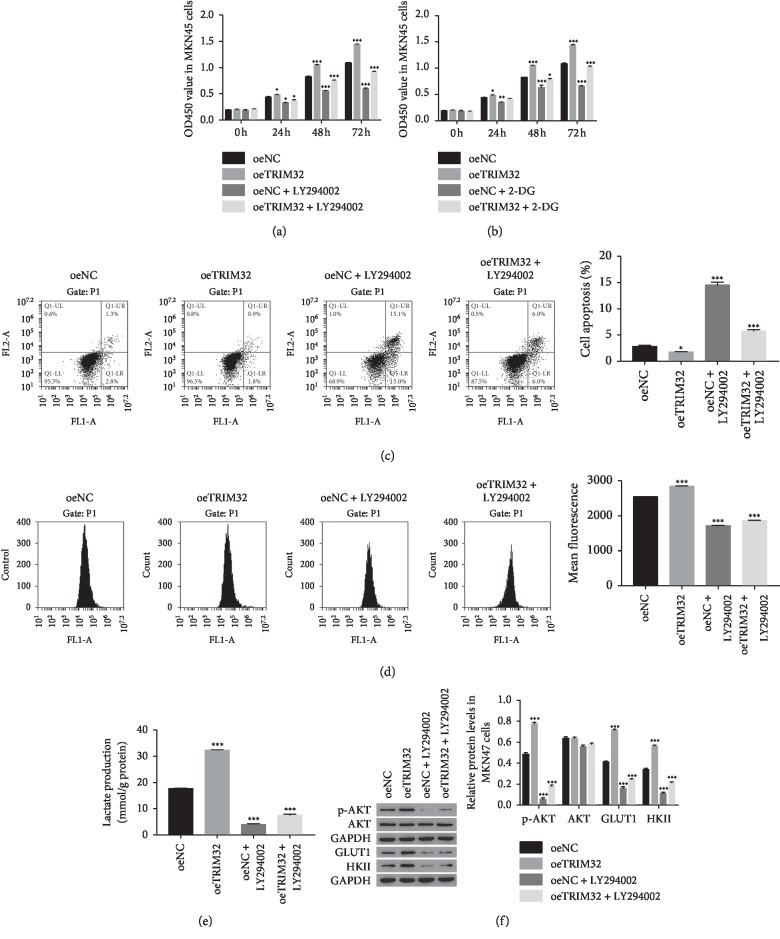
The function of TRIM32 was suppressed by the P13/AKT inhibitor LY294002. (a) Cell proliferation was detected 0, 24, 48, and 72 hours after transfection with oeNC, oeTRIM32, oeNC + LY294002, and oeTRIM32 + LY294002 in MKN45 cells, respectively. ^*∗*^*P* < 0.05 vs. oeNC, ^*∗∗∗*^*P* < 0.001 vs. oeNC. (b) Cell proliferation was detected 0, 24, 48 and 72 hours after transfection with oeNC, oeTRIM32, oeNC + 2-DG, and oeTRIM32 + 2-DG in MKN45 cells, respectively. ^*∗*^*P* < 0.05 vs. oeNC, ^*∗∗*^*P* < 0.01 vs. oeNC, ^*∗∗∗*^*P* < 0.001 vs. oeNC. (c) The apoptosis profile of oeNC, oeTRIM32, oeNC + LY294002, and oeTRIM32 + LY294002 in MKN45 cells, respectively. ^*∗*^*P* < 0.05 vs. oeNC, ^*∗∗∗*^*P* < 0.001 vs. oeNC. (d) Glucose transport activity measured using the fluorescent glucose analog 2-NBDG in MKN45 cells transfected with oeNC, oeTRIM32, oeNC + LY294002, and oeTRIM32 + LY294002. ^*∗∗∗*^*P* < 0.001 vs. oeNC. (e) The production of lactate in MKN45 cells transfected with oeNC, oeTRIM32, oeNC + LY294002, and oeTRIM32 + LY294002. ^*∗∗∗*^*P* < 0.001 vs. oeNC. ^*∗∗∗*^*P* < 0.001 vs. oeNC. (f). The protein level of AKT, p-AKT, GLUT1, and HKII in MKN45 cells transfected with oeNC, oeTRIM32, oeNC + LY294002, and oeTRIM32 + LY294002. ^*∗∗∗*^*P* < 0.001 vs. oeNC.

In order to further examine the correlation between AKT and TRIM32, the oeNC and oeTRIM32 transfected cells were cultured in the presence of a specific AKT inhibitor LY294002. As shown in Figure 5(a), the cell proliferation rate was obviously increased in oeTRIM32 transfected cells compared with oeNC. After being cultured with the inhibitor LY29004, cell proliferation rate was deeply suppressed in oeNC or oeTRIM32 transfected cells. Meanwhile, a glycolysis inhibitor 2-deoxyglucose (2-DG) was used to further determine the function of TRIM32 in cell proliferation. As shown in Figure 5(b), the cell proliferation rate of oeNC or oeTRIM32 transfected cells was also significantly inhibited by 2-DG. Therefore, these results indicated the similar function of the inhibitor LY294002 and 2-DG in the regulation of cell proliferation. Moreover, overexpression of TRIM32 significantly inhibited the apoptosis of GC cells, whereas this function was totally released by the AKT inhibitor LY29004 (Figure 5(c)). Furthermore, the AKT inhibitor LY294002 also significantly inhibited the production of glucose and lactate in oeTRIM32 transfected cells (Figures 5(d) and 5(e)). Additionally, the protein level of AKT, p-AKT, GLUT1, and HKII was also examined in different OE transfected cells. As shown in Figure 5(f), the phosphorylation of AKT was deeply suppressed by the inhibitor LY294002 in oeNC and oeTRIM32 transfected cells. Moreover, TRIM32 overexpression improved the phosphorylation of AKT in MKN45 cells in a time-dependent manner in the presence of the inhibitor LY294002 ([Supplementary-material supplementary-material-1]). Furthermore, the glycolysis-related protein GLUT1 and HKII were also significantly decreased by the inhibitor LY294002 in oeNC or oeTRIM32 transfected cells. Therefore, these results demonstrated that TRIM32 was involved in the AKT signaling pathway and regulated the glycolysis metabolism through targeting GLUT1 and HKII in GC cells.

### 3.8. TRIM32 Silencing Suppressed the Tumorigenicity of GC Cells *In Vivo*

**Figure 6:**
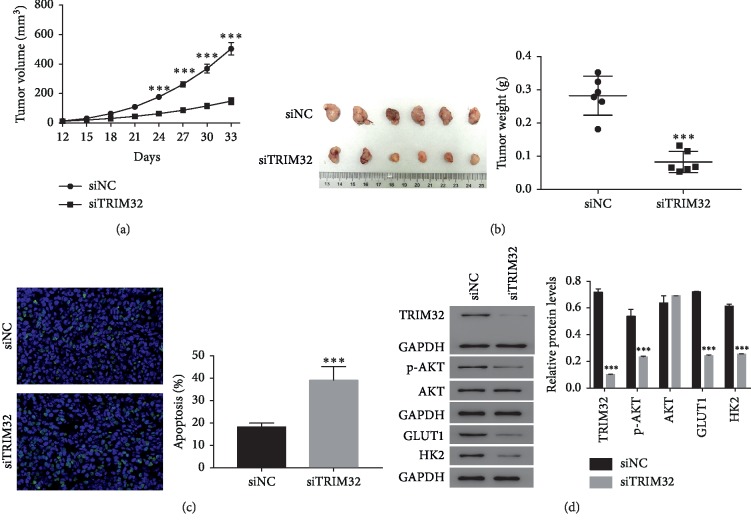
TRIM32 silencing suppressed the tumorigenicity of GC cells *in vivo*. (a) and (b) The tumor volume and weight were downregulated in nude mice that were injected with siTRIM32-transfected NCI-N87 cells. ^*∗∗∗*^*P* < 0.001 vs. siNC. (c) Tunnel staining assay was performed to examine the apoptosis ratio in siNC or siTRIM32 tumor, respectively. ^*∗∗∗*^*P* < 0.001 vs. siNC. (d) Western blot was used to examine the protein contents of TRIM32, p-AKT, AKT, GLUT1, and HK2 in siNC or siTRIM32 tumor, respectively. ^*∗∗∗*^*P* < 0.001 vs. siNC.

To further assess the role of TRIM32 in tumorigenicity *in vivo*, a number of 5 × 10^6^ of NCI-N87 cells that are transfected with siNC or siTRIM32 was hypodermically injected into nude mice (*n* = 6 for each group) and tumor formation was determined every 3 days for 33 days (starting at day 12). In the current study, both siNC and siTRIM32 transfected cells were capable to develop tumor *in vivo*. However, siTRIM32 cells significantly reduced the tumor volume as that of siNC cells. Moreover, the tumor weight of siTRIM32-injected mice was much lower than that in siNC-injected mice (Figures 6(a) and 6(b)). Furthermore, results collected from TUNEL assay indicated that siTRIM32 transfected cells remarkably improved the apoptosis rate of GC cells *in vivo* (Figure 6(c)). Together, all these results demonstrated that TRIM32 silencing deeply reduced the tumorigenicity of GC cells *in vivo*. Furthermore, the protein contents of TRIM32, p-AKT, GLUT1, and HK2 was deeply reduced in siTRIM32 tumors (Figure 6(d)).

## 4. Discussion

GC is one of the common death-related cancers all over the world. Although more and more attentions have been paid on improving its therapeutic strategies, the 5-year survival rate is still less than 30% [22]. Therefore, the novel effective treatment for GC is urgently needed. A previous report has indicated that TRIM32 overexpression correlates with poor prognosis in GC patients [23]. In the present research, we investigated the biological function of TRIM32 in GC cells. Our results indicated that TRIM32 was a proproliferation and antiapoptosis factor in GC cells. Therefore, these findings demonstrated that TRIM32 was an oncogene in the progression of human GC. More importantly, targeting TRIM32 might be a novel approach in the treatment for GC.

Previous report has reported that TRIM14 mediates cell proliferation in osteosarcoma though upregulating the AKT signaling pathway [24]. Moreover, the oncogene TRIM27 has activated the phosphorylation of AKT in colorectal cancer cells [25]. Recently, TRIM11 promotes proliferation and glycolysis of breast cancer cells via targeting the AKT/GLUT1 pathway [26]. Recently, TRIM32 is reported to promote cell proliferation and invasion in gastric cancer by activating *β*-catenin signalling [27]. Importantly, AKT-dependent regulation of *β*-catenin plays a critical role in tumor development [28]. In this study, we found TRIM32 was positively correlated with the phosphorylation of AKT in GC cells. Moreover, AKT inhibitor LY294002 blocked the function of TRIM32 in GC cells. To our knowledge, it was the first time to illustrate that the connection between TRIM32 and AKT in GC cells. Moreover, TRIM32 might promote the progression of human GC through regulating the phosphorylation of AKT.

Suppressing GLUT1 and HKII inhibits the activity of glycolysis and induces cell apoptosis [21, 29–31]. Moreover, the inactivation of the AKT-GLUTI/HKII signaling pathway suppressed the proliferation and glycolysis of lung cancer cells [32]. In the present research, our results firstly indicated the function of TRIM32 in the glycolysis metabolism in GC cells. Furthermore, TRIM32 might be a novel component in the AKT-GLUTI/HKII signaling pathway in GC cells. Furthermore, it has been confirmed that the combined inhibitors of AKT and glycolysis benefit lung cancer as indicated above [17]. Our results further provided evidences to indicate the potential value of the combination of the AKT inhibitor LY294002 and 2-DG in the treatment for GC.

## 5. Conclusion

In the present study, we systematically identified the function of TRIM32 in GC cells. Our findings not only indicated that the function of TRIM32 was achieved in GC cells might be via regulating the activity of AKT and glycolysis but also highlighted the potential value of TRIM32 for GC treatment.

## Data Availability

All data generated or analyzed during this study are included in this published article.

## Supplementary Materials

Supplementary Materials Supplementary Table 1: human gene TRIM32 (NM_012210.3) RNAi targeting locus information. Supplementary Table 2: the primary antibodies information. Figure S1: TRIM32 siRNAs inhibited the phosphorylation of AKT in a time-dependent manner in GC cells. A. Western blot was used to examine the protein contents of p-AKT and AKT in NC1-N87 cells that were transfected with siTRIM32-1 and siTRIM32-2 at 12, 24, and 48 h, respectively. B. Western blot was used to examine the protein contents of p-AKT and AKT in MKN74 cells that were transfected with siTRIM32-1 and siTRIM32-2 at 12, 24, and 48 h, respectively. Figure S2: overexpression of TRIM32 improved the phosphorylation of AKT in MKN45 cells in the presence of the 8 inhibitor LY294002.
