# Supplementary material for: TRIM32 Promotes the Growth of Gastric Cancer Cells through Enhancing AKT Activity and Glucose Transportation
Source: Biomed Res Int. 2020 Jan 21;2020:4027627. doi: 10.1155/2020/4027627 (PMC6995489; doi:10.1155/2020/4027627)
Supplement: Supplementary Materials — Supplementary Table 1: human gene TRIM32 (NM_012210.3) RNAi targeting locus information. Supplementary Table 2: the primary antibodies information. Figure S1: TRIM32 siRNAs inhibited the phosphorylation of AKT in a time-dependent manner in GC cells. A. Western blot was used to examine the protein contents of p-AKT and AKT in NC1-N87 cells that were transfected with siTRIM32-1 and siTRIM32-2 at 12, 24, and 48 h, respectively. B. Western blot was used to examine the protein contents of p-AKT and AKT in MKN74 cells that were transfected with siTRIM32-1 and siTRIM32-2 at 12, 24, and 48 h, respectively. Figure S2: overexpression of TRIM32 improved the phosphorylation of AKT in MKN45 cells in the presence of the 8 inhibitor LY294002. [file 4027627.f1.zip › 4027627.f1/mat.4027627.v2.docx]

# Supplementary File1: Primer sequence information

# 1.1 Homo sapiens tripartite motif containing 32 (TRIM32), transcript variant 1, mRNA

NM_012210.3

Primer F 5' GTGTCCGCTGTCCCTTTTG 3'

Primer R 5' GGCTGATGGTCTGCCTCCC 3'

Pos: 173-389

Amplified product: Size: 217 bps

**1.2 Homo sapiens glyceraldehyde-3-phosphate dehydrogenase (GAPDH), transcript variant 2, mRNA**

NM_001256799.1

Primer F 5' CACCCACTCCTCCACCTTTG 3'

Primer R 5' CCACCACCCTGTTGCTGTAG 3'

Pos: 1065-1174

Amplified product: Size: 110 bps

**Supplementary Table1: Human gene TRIM32 (NM_012210.3) RNAi targeting locus information**

| RNAi Targeting Locus | | Sequence |
| --- | --- | --- |
| Name | locus positon |  |
| RNAi1-1 | 499-517 | GGAGCTGTGGTTTGGTGTT |
| RNAi1-2 | 902-920 | GCAGGCAGATGTAGCACTA |
| RNAi1-3 | 1629-1647 | GGTGGAAAGCTTTGGTGTT |

**Supplementary Table 2: The primary antibodies information**

| Antibody name | Source | Dilution factor |
| --- | --- | --- |
| TRIM32 | Abcam, UK | 1:1000 |
| GLUT1 | Abcam, UK | 1:1000 |
| HKⅡ | CST, USA | 1:1000 |
| AKT | CST, USA | 1:1000 |
| P-AKT | CST, USA | 1:2000 |
| GAPDH | CST, USA | 1:1000 |
