# Supplementary material for: TRIM32 Promotes the Growth of Gastric Cancer Cells through Enhancing AKT Activity and Glucose Transportation
Source: Biomed Res Int. 2020 Jan 21;2020:4027627. doi: 10.1155/2020/4027627 (PMC6995489; doi:10.1155/2020/4027627)
Supplement: Supplementary Materials — Supplementary Table 1: human gene TRIM32 (NM_012210.3) RNAi targeting locus information. Supplementary Table 2: the primary antibodies information. Figure S1: TRIM32 siRNAs inhibited the phosphorylation of AKT in a time-dependent manner in GC cells. A. Western blot was used to examine the protein contents of p-AKT and AKT in NC1-N87 cells that were transfected with siTRIM32-1 and siTRIM32-2 at 12, 24, and 48 h, respectively. B. Western blot was used to examine the protein contents of p-AKT and AKT in MKN74 cells that were transfected with siTRIM32-1 and siTRIM32-2 at 12, 24, and 48 h, respectively. Figure S2: overexpression of TRIM32 improved the phosphorylation of AKT in MKN45 cells in the presence of the 8 inhibitor LY294002. [file 4027627.f1.zip › 4027627.f1/Supplementary Figures.pdf]

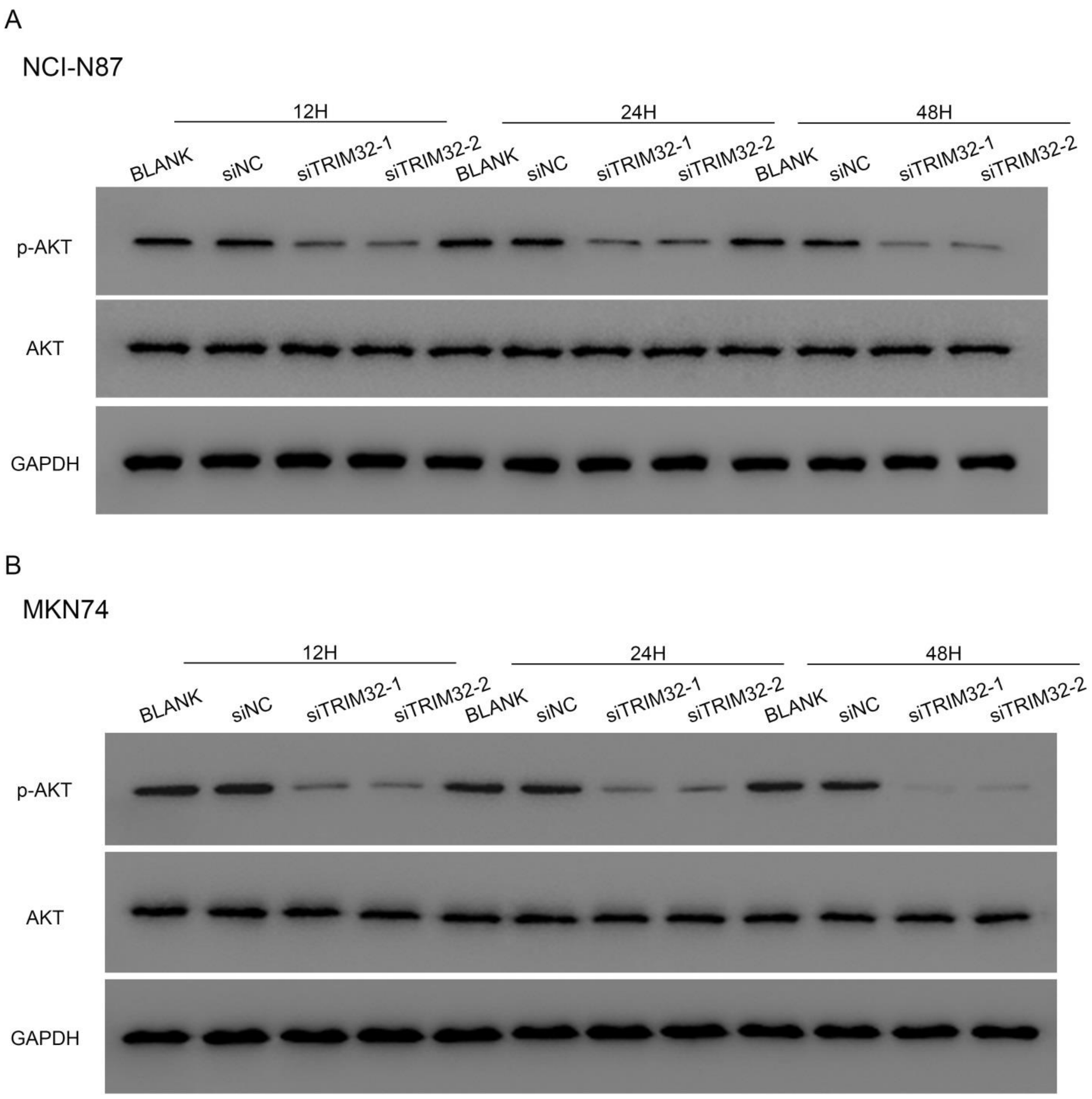

Figure S1: TRIM32 siRNAs inhibited the phosphorylation of AKT in a time-dependent manner in GC cells. A. Western blot was used to examine the protein contents of p-AKT and AKT in NC1-N87 cells that transfected with siTRIM32-1 and siTRIM32-2 at 12, 24 and 48h respectively. B. Western blot was used to examine the protein contents of p-AKT and AKT in MKN74 cells that transfected with siTRIM32-1 and siTRIM32-2 at 12, 24 and 48h respectively.

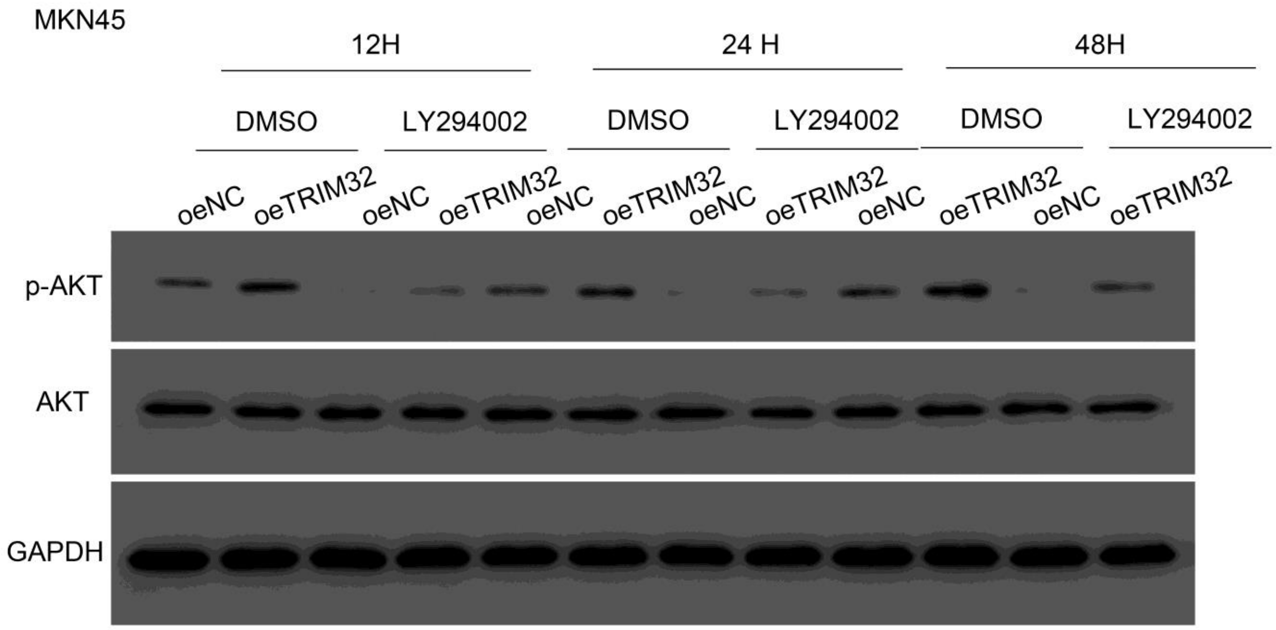

Figure S2: Overexpression of TRIM32 improved the phosphorylation of AKT in MKN45 cells in the presence of the inhibitor LY294002.
